# Abnormal focal segments in left uncinate fasciculus in adults with obsessive–compulsive disorder

**DOI:** 10.3389/fpsyt.2023.1128808

**Published:** 2023-03-30

**Authors:** Suming Zhang, Bin Li, Jiaxin Jiang, Xinyu Hu, Hailong Li, Lingxiao Cao, Zilin Zhou, Kaili Liang, Huan Zhou, Lianqing Zhang, Qiyong Gong, Xiaoqi Huang

**Affiliations:** ^1^Huaxi MR Research Center (HMRRC), Functional and Molecular Imaging Key Laboratory of Sichuan Province, Department of Radiology, West China Hospital, Sichuan University, Chengdu, China; ^2^Psychoradiology Research Unit of the Chinese Academy of Medical Sciences, West China Hospital of Sichuan University, Chengdu, Sichuan, China; ^3^Mental Health Centre, West China Hospital, Sichuan University, Chengdu, Sichuan, China; ^4^Department of Radiology, West China Xiamen Hospital of Sichuan University, Xiamen, Fujian, China

**Keywords:** obsessive–compulsive disorder, diffusion tensor imaging, automated fiber quantification, uncinate fasciculus, insula

## Abstract

**Background:**

Although the specific role of the uncinate fasciculus (UF) in emotional processing in patients with obsessive–compulsive disorder (OCD) has been investigated, the exact focal abnormalities in the UF have not been identified. The aim of the current study was to identify focal abnormalities in the white matter (WM) microstructure of the UF and to determine the associations between clinical features and structural neural substrates.

**Methods:**

In total, 71 drug-naïve patients with OCD and 81 age- and sex-matched healthy controls (HCs) were included. Automated fiber quantification (AFQ), a tract-based quantitative approach, was adopted to measure alterations in diffusion parameters, including fractional anisotropy (FA), mean diffusivity (MD), radial diffusivity (RD) and axial diffusivity (AD), along the trajectory of the UF. Additionally, we utilized partial correlation analyses to explore the relationship between the altered diffusion parameters and clinical characteristics.

**Results:**

OCD patients showed significantly higher FA and lower RD at the level of the temporal and insular portions in the left UF than HCs. In the insular segments of the left UF, increased FA was positively correlated with the Hamilton Anxiety Scale (HAMA) score, while decreased RD was negatively correlated with the duration of illness.

**Conclusion:**

We observed specific focal abnormalities in the left UF in adult patients with OCD. Correlations with measures of anxiety and duration of illness underscore the functional importance of the insular portion of left UF disturbance in OCD patients.

## 1. Introduction

Obsessive–compulsive disorder (OCD) has a lifetime prevalence of 1–3% worldwide and is characterized by compulsions or obsessions that generate fear and anxiety (with or without autonomic symptoms) or doubts/uncertainty (1). The pathophysiological mechanism of OCD is unclear. In addition to the traditional cortico-striatal-thalamo-cortical (CSTC) circuit (2–4), studies have proven that the fronto-limbic system plays a vital role in emotional regulation among OCD patients (5, 6). Dysregulated fear and uncertainty intolerance aggravate obsession and are closely related to abnormalities of the fronto-limbic circuit in OCD (7).

The hook-shaped uncinate fasciculus (UF) is a white matter bundle belonging to the fronto-limbic circuit, which connects the lateral orbitofrontal cortex and the prefrontal cortex to the anterior temporal lobes and basolateral amygdala (8, 9). The UF plays a putative role in social emotional processing and episodic memory (10), which are impaired in OCD patients (11). Diffusion tensor imaging (DTI) allows the quantification of white matter microstructure and can reveal the brain substrates of pathologic alterations in structural connectivity (12). Examination of the abnormal microstructure of key white matter tracts in individuals with OCD can improve our understanding of the neural mechanisms that underlie this disorder.

Recent studies have elucidated abnormalities related to the UF using DTI techniques in OCD patients both at the group level (13–15) and at the individual level (16). At the group level, one study employing probabilistic tractography confirmed a decrease of fractional anisotropy (FA) in the right UF in OCD patients in addition to a significant correlation between axial diffusivity (AD) in the right UF and symptom severity (14). At the individual level, a multimodality study that combined whole-brain volumetry and diffusion tensor imaging used a support vector machine (SVM) to discriminate between OCD patients and HCs (16). The UF was identified as the region that contributed the most to this discriminatory ability (16). Taken together, this evidence indicates that the UF plays a potential role in the neuropathologic mechanisms of OCD.

Previous studies utilizing voxel-based analyses or tract-based spatial statistics (TBSS) revealed that the UF showed decreased integrity in OCD (14). Although those whole-brain studies provided evidence that some symptoms of OCD might be linked to the integrity of the UF, no previous study has provided a detailed description of the abnormalities along the trajectory of the UF in OCD patients. Thus, in the current study, we assessed the bilateral UF by quantifying diffusion parameters at multiple nodes along the tract’s trajectory rather than obtaining the mean value of the whole fiber using an automatic fiber quantification (AFQ) method similar to our previous study (17). We hypothesized that specific focal regions in the UF would be more vulnerable to microstructural dysconnectivity and correlated with symptom severity in patients with OCD.

## 2. Materials and methods

### 2.1. Participants

The study was approved by the Institutional Reviews Board of West China Hospital, Sichuan University. After being informed of the nature and aims of the study, all subjects signed a consent form before participating in the study procedures.

Seventy-one adults with OCD (age range = 18–35 years, mean age = 30.14 years; male percentage = 60.83%) and 81 age- and sex-matched healthy adult controls (age range = 18–37 years, mean age = 29.76 years; male percentage = 58.33%) were included in this study. All patients were right handedness and native Han Chinese. Patients were recruited at the West China Hospital, Sichuan University. All OCD patients had never received medication or systematic psychotherapy before MRI data acquisition. We established a clinical diagnosis using the Structured Clinical Interview for DSM-IV Axis I disorders (SCID) (18), which was administered by two experienced clinical psychiatrists. We assessed the severity of OCD symptoms using the Yale-Brown Obsessive–Compulsive Scale (19). The 14-item Hamilton Anxiety Rating (HAMA) Scale (20) and 17-item Hamilton Depression Rating (HAMD) Scale (21) were used to assess accompanying anxiety and depression.

Healthy controls were recruited from the local area using poster advertisements and screened using the SCID (non-patient version) to confirm the current absence of psychiatric disorders, as well as the absence of a history of psychiatric disorders among their first-degree relatives.

The exclusion criteria, applied to both OCD patients and HCs, were as follows: age younger than 18 years or older than 60 years; history of a psychotic, affective, or anxiety disorder other than OCD, as determined with the SCID; a history of significant systemic illness, cardiovascular disease, or neurologic disorder; substance abuse or dependence; and pregnancy.

### 2.2. Data acquisition

Images were acquired on a 3 T GE (EXCITE, General Electric) magnetic resonance imaging (MRI) system with an eight-channel phased array head coil. A single-shot spin echo planar imaging (EPI) sequence was acquired with 15 noncolinear directions (*b* = 1000 s/mm^2^) and one reference volume with *b* = 0 s/mm^2^. Repetition time (TR) = 12,000 ms, echo time (TE) = 70.8 ms, slice thickness = 3 mm (no slice gap), number of excitations = 2, matrix = 128*128, field-of-view (FOV) = 240 * 240 mm^2^, and voxel size = 1.875*1.875*3 mm^3^. DTI was performed using axial sections parallel to the anterior–posterior commissural line to cover the entire brain. As an anatomical reference for normalization, high-resolution T1-weighted images were acquired using a 3D spoiled gradient recalled (SPGR) sequence (TR/TE = 8.5/3.4 ms, 156 slices with thickness 1 mm, flip angle = 12°, matrix = 256 * 256, FOV = 240 * 240 mm^2^, and voxel size = 0.93 * 0.93 * 1 mm^3^).

### 2.3. Data preprocessing and quality assurance

DTI data were preprocessed by using the FMRIB Software Library (FSL) 6.0.^1^ All data were visually inspected for artifacts. Brain extraction was completed *via* FSL’s “BET” function (22) and correction for the effects of head motion and image distortions caused by eddy currents was performed using “EDDY” tool (23–26). FA images were created by fitting a tensor model to the raw diffusion data using the FMRIB Diffusion Toolbox (FDT) (27). The high-resolution T1-weighted brain structural images were coregistered into the averaged b0 images for each subject. We extracted the head motion parameters from DTI data to exclude subjects showing >2 mm displacement or translation in the X, Y, and Z directions or >2° rotation around the X, Y, Z axes (28, 29); the motion measurements did not differ between the OCD group and HC group (details in Supplementary Table S1).

### 2.4. AFQ tractography

Automated deterministic tractography of bilateral UF was performed according to standard protocols and pipelines, which are described by Yeatman in detail (30). Reconstruction of UF was completed *via* the open-source VISTASOFT package version 1.0^2^ and AFQ toolkit package^3^ (version 1.2) (30).

First, whole-brain deterministic fiber tractography was estimated. The tracking algorithm started within a WM mask defined by voxels with FA values >3; then, continuing the path integration procedure, the fibers were traced in both directions along principal diffusion axes. Tracing was terminated when FA < 2 or the minimum angle between the last path segment and next step was >30. Fiber tract segmentation was performed using the waypoint ROI procedure, and fiber refinement was accomplished by comparing each candidate fiber to fiber tract probability maps. After the bilateral UF tracts were identified, the fiber points of each tract were resampled by using 100 equidistant points, and then the diffusion measurements of each participant, including the FA, MD, RD and AD, were extracted along the UF (15).

### 2.5. Statistical analysis

Two-sample *t*-tests were carried out to evaluate differences in DTI parameters (FA, MD, AD, and RD) between the OCD group and HC group at each node in the bilateral UF. Afterward, partial correlation analyses were further performed between symptom severity and DTI parameters (FA, MD, AD, and RD) extracted from significant nodes in the bilateral UF, with age and sex as covariates. Significant results were corrected for multiple comparisons using the false discovery rate (FDR corrected *p*-value < 0.05).

## 3. Results

### 3.1. Participant characteristics

The demographic and clinical characteristics of the participants in this study are shown in Table 1.

**Table 1:** Demographics and clinical characteristics of enrolled subjects.

Items	OCD group (*N* = 71)	HC group (*N* = 81)	*t*/*χ*^2^	Value of *p*
Age (mean ± SD)	30.14 ± 8.9	29.76 ± 10.7	0.44	0.66
Sex (male:female)	48:23	42:39	2.33	0.94
Illness duration (years)	7.68 ± 5.71	–	–	–
Illness onset age	22.77 ± 7.88			
YBOCS	21.66 ± 5.46			
Obsession	13.12 ± 5.19	–	–	–
Compulsion	8.57 ± 5.40	–	–	–
HAMA	9.23 ± 4.84	–	–	–
HAMD	8.73 ± 5.24	–	–	–

OCD, obsessive–compulsive disorder; HC, healthy control; SD, standard deviation; YBOCS, Yale-Brown Obsessive–Compulsive Scale; HAMD, Hamilton Depression Rating Scale; HAMA, Hamilton Anxiety Rating Scale.

### 3.2. Group comparison between the OCD and HC groups

**Figure 1:**
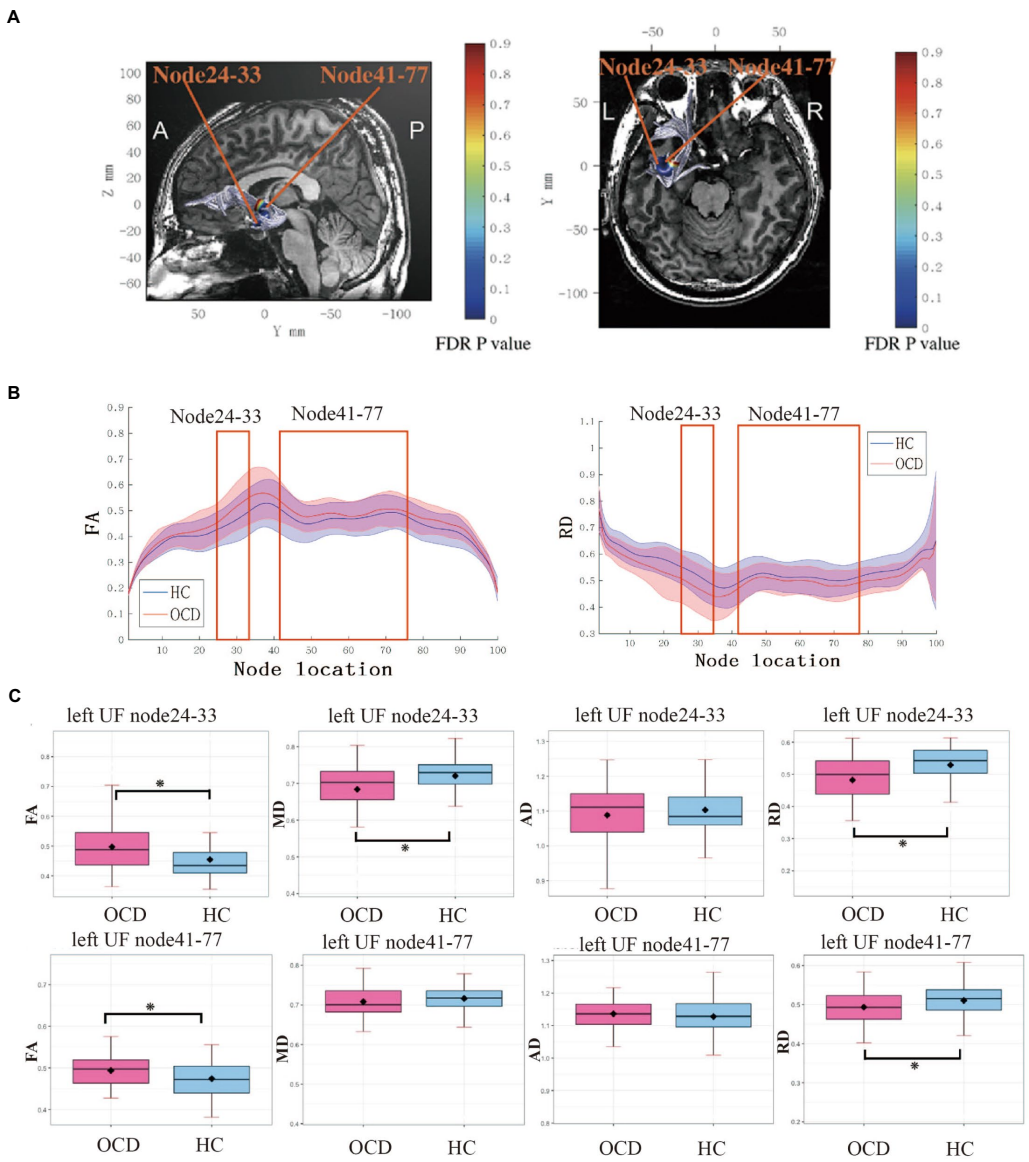
**(A)** Left UF tract with significant group differences (*p* < 0.05, FDR corrected) at two segments: temporal portion (node 24–33) and insular portion (node 41–77). **(B)** FA between nodes 1 and 100 for the left UF in OCD group and HC group. Solid lines represent the mean FA, and dotted lines denote the standard error of the mean. Consecutive nodes that showed significant differences are marked with red rectangles. (*p* < 0.05, FDR corrected). **(C)** Boxplots for FA, RD, MD, and AD in two significant segments between groups. Error bars represent standard deviation. UF, uncinate fasciculus; FA; fractional anisotropy; MD, mean diffusivity; AD, axial diffusivity, RD, radial diffusivity; OCD, obsessive–compulsive disorder; HC, healthy control.

In the point-wise comparison with HCs, significantly increased FA and reduced MD and RD were found at the level of the temporal portion (nodes 24–33) in the left UF tracts in OCD (*p* <0.05, FDR correction), while significantly increased FA and decreased RD were observed at the level of the insular segments (nodes 41–77) in the left UF tracts in OCD (*p* < 0.05, FDR correction; Figure 1).

### 3.3. Partial correlation between abnormal microstructure of the UF and symptom severity

**Figure 2:**
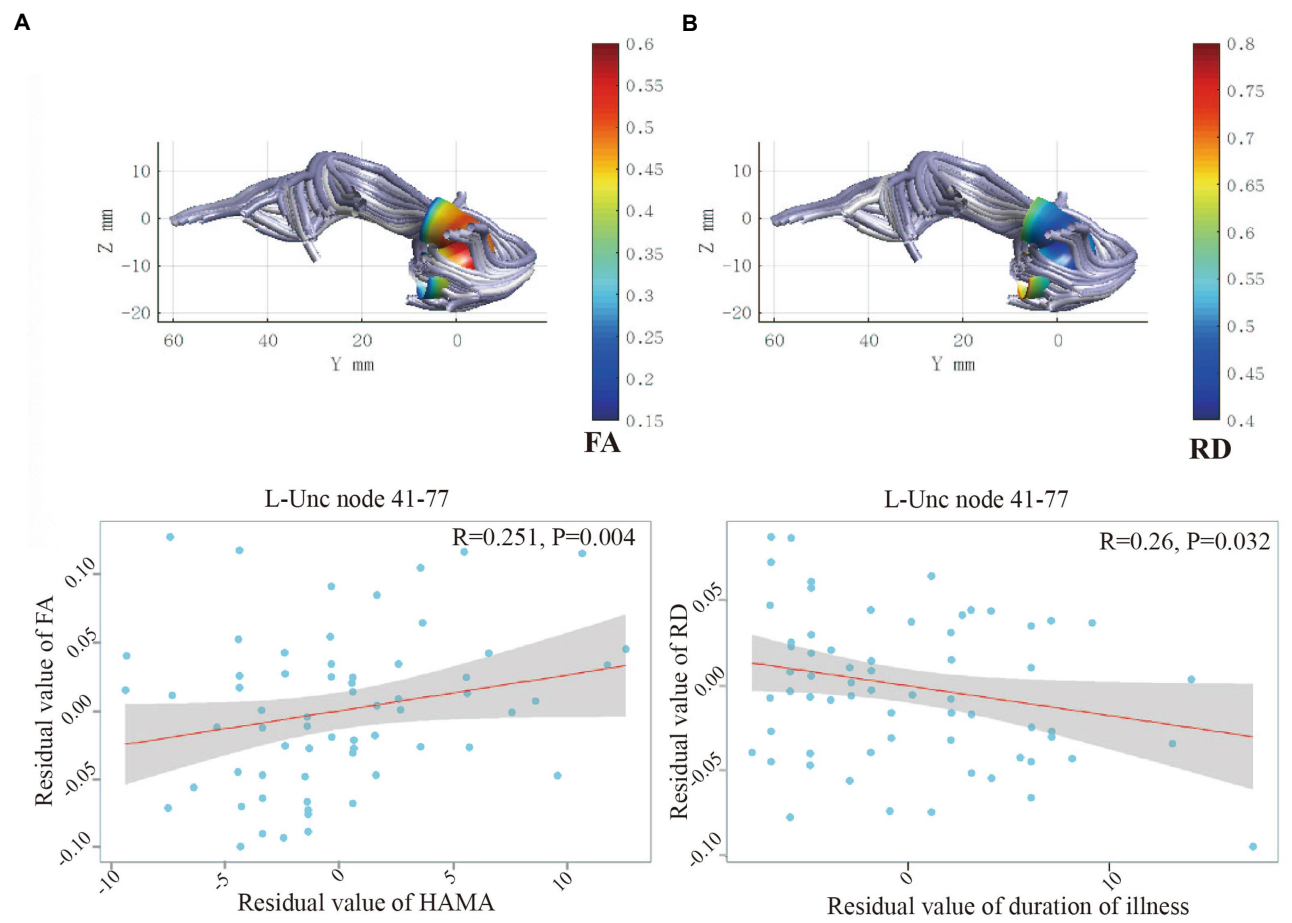
**(A)** Positive correlation between HAMA and FA for the node 41–77 of left UF. **(B)** Negative correlation between duration of illness and RD for the node 41–77 of left UF. UF, uncinate fasciculus; FA; fractional anisotropy; RD, radial diffusivity; OCD, obsessive–compulsive disorder; HC, healthy control; HAMA, Hamilton Anxiety Scale.

In OCD patients, the mean FA of nodes 41–77 of the left UF correlated positively with HAMA scores (*R* = 0.251, *p* = 0.004). In addition, the mean RD of nodes 41–77 correlated negatively with the duration of illness (*R* = −0.26, *p* = 0.032; Figure 2).

## 4. Discussion

To our knowledge, this is the first study utilizing a tractography method to explore the focal abnormality of the UF in individuals with OCD. We found significantly higher FA along with lower RD at the level of the temporal portion and insular portion of the left UF in OCD patients than in HCs. Within the focal insular portion of the left UF, the increased FA difference showed a positive correlation with anxiety severity in individuals with OCD, whereas the decreased RD showed a negative correlation with the duration of illness. Taken together, these findings elucidated the focal structural connectivity of the left UF in neural mechanisms of OCD, which may help to innovate more targeted interventions for individuals with OCD in the future.

The UF connects the lateral orbitofrontal cortex and the prefrontal cortex to the anterior temporal lobes and basolateral amygdala (3, 4). The hook-shaped fiber plays a putative role in social emotional processing (5). Additionally, it is a key component of the fronto-limbic network, which plays a significant role in OCD pathophysiology (31). Evidence suggests that higher FA together with lower RD is related to increased myelination, dense axonal packing, white matter maturation, and neuronal remodeling (32–34). Our finding of higher FA suggests that OCD patients may exhibit greater fiber integrity in the insular portion and temporal portion of the left UF relative to HCs. Similar to the interpretation of Li et al., an increase in FA might be a compensatory process for neuronal injury in individuals with OCD (35). The negative correlation between decreased RD and the duration of illness indicated that flawed WM microstructure has predictive ability for the duration of illness. The longer the duration of illness, the lower the RD of the insular part of the left UF. Our finding is different from that of another TBSS study (36). That study reported higher RD in the whole left UF and no finding of an association with the duration of illness in patients with OCD.

Compared to previous studies that reported abnormalities in the entire uncinate fasciculus (13–15, 36), we demonstrated that higher FA and lower RD are focal in the insular portion and temporal portion of the left UF in individuals with OCD. Furthermore, increased FA in the insular portion of the left UF showed a positive correlation with anxiety severity in our study. It can be inferred that the more severe the anxiety symptoms are, the greater the remyelination that occurs in the insular portion of the left UF among OCD patients based on our findings. Evidence has shown that the insular lobe might be involved in affective processing (37). In particular, the insular cortex is believed to be involved in OCD (38–41). Our findings add evidence that abnormal fiber integrity in the insular portion of the left UF may underlie anxiety symptoms in individuals with OCD. Furthermore, decreased gray matter volume in the insular region has been reported in a voxel-based morphometric study among OCD patients (42). Thus, it may be inferred that greater myelination in the insular segment of the left UF, which increases the speed of information communication, is parallel to abnormal gray matter volume in the insular cortex area among OCD patients.

Our findings in the UF of OCD patients are different from those of previous diffusion MRI studies in OCD patients. In contrast to abnormal fiber integrity in the left UF in our findings, one former study reported a lower FA and higher RD of the right UF in OCD patients than in HCs (14, 36). We inferred that these differences are due to the analysis method and patient status. The TRActs Constrained by UnderLying Anatomy (TRACULA), which belongs to probabilistic tractography, was used in the former studies (14, 36), whereas deterministic tractography was utilized in our study. Another previous TBSS study demonstrated increased RD and MD in the bilateral UF in individuals with OCD (36), and some potential reasons for these differences in findings may be attributable to variations in the sample size, patient medication status and MRI scan parameters.

Our study had several limitations. First, we did not employ pediatric OCD patients in the current investigation. A previous publication identified distinct WM alterations in pediatric and adult OCD (43). Future studies involving pediatric OCD patients are needed to shed light on the neurodevelopmental alterations of WM in individuals with OCD. Second, since we included only medication-naïve patients with OCD, it remains to be explored whether our results could be generalized to larger OCD populations undergoing long-term treatment.

In summary, the present study revealed abnormal white matter changes along the left UF tract using a tract-wise approach in individuals with OCD. We demonstrated that altered fiber integrity is focal in the insular and temporal portions of the left UF. The increased FA and decreased RD of the insular part of the left UF are associated with anxiety and duration of illness, respectively. Our study suggests that the insular portion of the left UF plays an important role in OCD pathophysiology.

## Data availability statement

The original contributions presented in the study are included in the article/supplementary material, further inquiries can be directed to the corresponding author/s.

## Ethics statement

The studies involving human participants were reviewed and approved by the Institutional Review Board of the West China Hospital, Sichuan University. The patients/participants provided their written informed consent to participate in this study.

## Author contributions

SZ, XiaoqiH, and QG designed the study. BL, JJ, and XinyuH acquired the data. SZ, KL, and LZ analyzed the data. SZ, BL, and XinyuH wrote the article. HL, LC, ZZ, HZ, and XiaoqiH reviewed the article. All authors contributed to the article and approved the submitted version.

## Funding

The authors would like to thank their tutor and colleagues for their time and valuable help. This study was supported by the Clinical and Translational Research Fund of Chinese Academy of Medical Sciences (grant no. 2021-I2M-C&T-B-097), the Natural Science Foundation of Sichuan Province (grant no. 2022NSFSC0052), and the 1·3·5 Project for Disciplines of Excellence, West China Hospital of Sichuan University (grant no. ZYJC21041).

## Conflict of interest

The authors declare that the research was conducted in the absence of any commercial or financial relationships that could be construed as a potential conflict of interest.

## Publisher’s note

All claims expressed in this article are solely those of the authors and do not necessarily represent those of their affiliated organizations, or those of the publisher, the editors and the reviewers. Any product that may be evaluated in this article, or claim that may be made by its manufacturer, is not guaranteed or endorsed by the publisher.

## Footnotes

^1^ https://fsl.fmrib.ox.ac.uk/fsl/

^2^ https://github.com/vistalab/vistasoft

^3^ https://github.com/yeatmanlab/AFQ
