# Supplementary material for: Abnormal focal segments in left uncinate fasciculus in adults with obsessive–compulsive disorder
Source: Front Psychiatry. 2023 Mar 30;14:1128808. doi: 10.3389/fpsyt.2023.1128808 (PMC10098161; doi:10.3389/fpsyt.2023.1128808)
Supplement: Supplementary file 1 [file Table_1.DOCX]

***Supplementary Materials***

**Abnormal focal segments in left uncinate fasciculus in adults with obsessive-compulsive disorder**

Suming Zhang^1,2,^^†^, Bin Li^3,†^, Jiaxin Jiang^3^, Xinyu Hu^1,2^, Hailong Li^1,2^, Lingxiao Cao^1,2^, Zilin Zhou ^1,2^, Kaili Liang^1,2^, Huan Zhou^1,2^, Lianqing Zhang^1,2^, Qiyong Gong^1,4,*^ and Xiaoqi Huang^1,2,*^

^*^**Corresponding authors：**

Xiaoqi Huang, Huaxi MR Research Center (HMRRC), Department of Radiology, West China Hospital of Sichuan University, No. 37 Guo Xue Xiang, Chengdu, 610041, Sichuan Province, China. Tel: 86-28-85423255, Fax: 86-28-85423503. Email:  [julianahuang@163.com](mailto:julianahuang@163.com).

Qiyong Gong, Department of Radiology, West China Xiamen Hospital of Sichuan University, 699 Jinyuan Xi Road, Jimei District，361021 Xiamen, Fujian, China. Email: qiyonggong@hmrrc.org.cn.

Supplementary Table

**Table S1. Head movement metrics comparison between OCD and HC group**

| **Measurements** | **OCD group** | **HC group** | **P value** |
| --- | --- | --- | --- |
| Relative displacement | 0.19±0.0475 | 0.26±0.1444 | 0.31 |
| Rotation around X (degree) | -0.0006±0.125 | 0.037±0.1915 | 0.14 |
| Rotation around Y (degree) | 0.0058±0.058 | 0.0247±0.099 | 0.15 |
| Rotation around Z (degree) | -0.00948±0.009 | -0.0087±0.0947 | 0.96 |
| Translation along X (mm) | -0.015±0.0557 | -0.022±0.1365 | 0.70 |
| Translation along Y (mm) | 0.097±0.0677 | 0.1112±0.0843 | 0.24 |
| Translation along Z (mm) | -0.038±0.1592 | 0.011±0.2102 | 0.96 |

[*Abbreviation*](javascript:;)*: OCD, obsessive compulsive disorder; HC, healthy control.*
